# Book Review: Such a time of it they had: Global health pioneers in Africa

**DOI:** 10.4102/phcfm.v12i1.2294

**Published:** 2020-01-16

**Authors:** Louis S. Jenkins

**Affiliations:** 1 Department of Family and Emergency Medicine, Division of Family Medicine and Primary Care, Stellenbosch University, Cape Town, South Africa; 2 Department of Health, George Regional Hospital, Garden Route, George, Western Cape, South Africa

This is one of those books that draws the reader in from the beginning, starting with the unusual title, and building an information-rich, honest, provocative and narrative-style story of global health, colonialism, conflict, fever and philosophy. Through the eyes of a seasoned medical doctor, Downing tells the stories of medical history focusing on global health, while not valorising or criticising its major figures. It is a memoir of two physicians and their 30 years of work in Africa, unpacking aspects of African history, showing how these aspects influenced their experiences long before they appeared on the scene.

Beginning with the Dutchman, Van der Kemp, Downing traces the history of western medical workers in Africa, from explorers such as Mungo Park and David Livingstone, through to Jane Waterston, the first woman physician in Southern Africa. What kind of doctors were they? ‘They were Romantics. They were only surgeons, upgraded barbers, they were not in the great tradition of healers. They were sent to explore, they had no mandate to further human health or reduce suffering. They were sick and tired. They were Europeans of their day, drawn to nature, to the exotic, to passion, to glorious death’. They lived in an age when the industrial revolution was being launched, when science promised far more than it could deliver to medicine.

The book helps the reader understand how these early doctors were also part of a global discourse. For example, during Livingstone’s time, the recently developed stethoscope, a ‘hollow tube without moving parts’, developed by Laennec (1819), represented a different way of approaching all of medicine, focusing on the body itself and its discontents. Until then the physical examination was optional. The patient’s story was paramount, as was the doctor’s interpretation of that story according to the humoral theory, the foundation of western medicine for two millennia. Livingstone was an early adopter of stethoscope and explored unchartered geographical territories as well as practicing medicine in the days before the germ theory.

Many terrible mistakes were made, and the author gives us a glimpse into some of their own blind spots. We learn how tropical medicine wants to ‘control’, connecting the dots between the British assault on the Ashanti Empire (1874), when quinine was still not understood but used extensively to keep the British soldiers alive, and the 2014 Ebola outbreak in west Africa, where the first three American global health missionary workers infected were given the latest experimental drug (ZMapp) for Ebola, and survived. This prompted a news article: ‘Faith, Medicine or ZMapp? What Cured the Ebola Patients?’

Unlike many medical histories, *Such a Time* does not trumpet successive ‘cures’ for malaria, sleeping sickness and even Ebola. Rather, it wishes to speak about global health as it relates to patients. What good are these ‘discoveries’ if the medical staff is ill-trained and hospitals lack basic facilities and medications? Early in the book, Downing laughs at himself and his peers of the time trying to choose a theme for a 1980s conference on underserved people. They first agreed upon service to others. Next, reflecting the time, they agreed to add solidarity, and finally empowerment. *Such a Time* reflects his appraisal of global health in Africa from the time of colonisation, and during his lifetime, from these slogans. ‘Empowerment was out of the hands of doctors, service was not enough; and no one seemed to know what solidarity was. Yet, despite all our “progress,” the individual who most closely achieved these was the very limited physician van der Kemp, who lived among and even married one of his African patients’.

Looking back over the more recent 10 years, they were hired directly by a Kenyan university to help develop a family medicine training programme. Juggling jobs between dealing with ‘expert’ donor funders and other expatriate do-gooders and doing routine ward rounds at 08:00 every morning, getting behind the reason a child with cerebral malaria is not yet waking up after three days in the ward, navigating the medicine, laboratory and care pathways, and taught them many things. ‘Ah, but such a time of it we’ve had. Not always a good time, but a full time certainly: a window into the time our forbearers had, earlier global health workers in Africa, long before we were calling this “global health.”’

From *Such a Time* we come to know a great deal about race, class, gender, power and even human psychology. But even more it is a book that first implicitly and finally explicitly questions the Eurocentric Enlightenment model of the spread of western religion, science and methods. Reflective of this, the title comes from the poem ‘Stanley Meets Mutesa’ by David Rubadiri, a noted Malawian man. While it may have been such a time, Downing succeeds at questioning from a very different time what it has all meant.

